# The Therapeutic Role of Exercise and Probiotics in Stressful Brain Conditions

**DOI:** 10.3390/ijms23073610

**Published:** 2022-03-25

**Authors:** Ismael Martínez-Guardado, Silvia Arboleya, Francisco Javier Grijota, Aleksandra Kaliszewska, Miguel Gueimonde, Natalia Arias

**Affiliations:** 1 BRABE Group, Department of Psychology, Faculty of Life and Natural Sciences, University of Nebrija, C/del Hostal, 28248 Madrid, Spain; fgrijota@nebrija.es; 2 Department of Microbiology and Biochemistry of Dairy Products, Instituto de Productos Lácteos de Asturias (IPLA-CSIC), 33003 Oviedo, Spain; silvia.arboleya@ipla.csic.es (S.A.); mgueimonde@ipla.csic.es (M.G.); 3 Department of Basic and Clinical Neuroscience, Institute of Psychiatry, Psychology and Neuroscience, King’s College London, Denmark Hill, London SE5 8AF, UK; aleksandra.kaliszewska@kcl.ac.uk; 4 Health Research Institute of the Principality of Asturias-ISPA, 33011 Oviedo, Spain; 5 Institute of Neurosciences of the Principality of Asturias (INEUROPA), 33003 Oviedo, Spain

**Keywords:** exercise, neurogenesis, oxidative stress, probiotics, brain, therapy

## Abstract

Oxidative stress has been recognized as a contributing factor in aging and in the progression of multiple neurological disorders such as Parkinson’s disease, Alzheimer’s dementia, ischemic stroke, and head and spinal cord injury. The increased production of reactive oxygen species (ROS) has been associated with mitochondrial dysfunction, altered metal homeostasis, and compromised brain antioxidant defence. All these changes have been reported to directly affect synaptic activity and neurotransmission in neurons, leading to cognitive dysfunction. In this context two non-invasive strategies could be employed in an attempt to improve the aforementioned stressful brain status. In this regard, it has been shown that exercise could increase the resistance against oxidative stress, thus providing enhanced neuroprotection. Indeed, there is evidence suggesting that regular physical exercise diminishes BBB permeability as it reinforces antioxidative capacity, reduces oxidative stress, and has anti-inflammatory effects. However, the differential effects of different types of exercise (aerobic exhausted exercise, anaerobic exercise, or the combination of both types) and the duration of physical activity will be also addressed in this review as likely determinants of therapeutic efficacy. The second proposed strategy is related to the use of probiotics, which can also reduce some biomarkers of oxidative stress and inflammatory cytokines, although their underlying mechanisms of action remain unclear. Moreover, various probiotics produce neuroactive molecules that directly or indirectly impact signalling in the brain. In this review, we will discuss how physical activity can be incorporated as a component of therapeutic strategies in oxidative stress-based neurological disorders along with the augmentation of probiotics intake.

## 1. Introduction

Physical activity (PA) is a modifiable lifestyle factor associated with improved overall health, reduced premature mortality, and a demonstrable role in the prevention of several chronic conditions [1,2,3]. Exercise promotes brain health by supporting brain’s structural integrity and function, which may improve cognitive performance and counteract at least certain aspects of cognitive ageing.

Several longitudinal studies have suggested that maintaining regular PA is linked to a reduced risk of cognitive impairments in older adults. A meta-analysis of 15 prospective studies has found that both high and moderate-low levels of exercise exert significant protection against cognitive decline in non-demented older individuals [4]. Notably, a recent meta-analysis has demonstrated that habitual PA prevents hippocampal volumetric decreases, which occur over time in humans [5]. The hippocampus is the primary centre of learning and memory, and since age-related hippocampal atrophy is a strong predictor of future cognitive decline [6,7,8], these findings hold promising implications for combating cognitive decline through exercise-induced retention of hippocampal volume.

Moreover, there is evidence to suggest that exercise may have a protective role against the development and progression of neurodegenerative disorders such as Parkinson’s disease (PD) and Alzheimer’s disease (AD). In a recent meta-analysis, Santos-Lozano and colleagues have found that adhering to the international PA guidelines of ≥150 min/week of moderate-intense activity was associated with a 40% reduction of risk for the development of AD [9]. PA was also shown to have cognition-enhancing effects in individuals at high risk of developing AD and in those affected by the disease [10,11,12]. Similarly, a systematic review evaluating the effect of exercise on cognitive function in PD has found evidence to support the role of PA in improving cognition in both animal models and human patients [13].

On the other hand, over the last two decades, the gut microbiota has emerged as an important organ with key functions such as the training of host immunity, digesting food, regulating gut endocrine, and neurological functions [14,15]. Evidence continues to demonstrate that the gut microbiota is particularly implicated in brain physiology and behavior, affecting host mental health [16,17,18]. New advances on the techniques for its analyses have allowed for knowing deeply the gut microbiome on different human conditions (age- or healthy-related) [19,20,21]; however, a “gold standard” reference of a human gut microbiota composition is not yet known. What is known is that the imbalance of the gut microbiota composition or functions can affect physiological homeostasis and alter the signals from the gut to brain, negatively influencing brain health or vice versa [22,23]. Different factors can be affect the gut microbiota, exercise being one of them [24]. Additionally, the modulation of the microbiota for improving cognition has attracted a deal of attention in recent years. The strategy most widely studied to this end is the use of probiotic microorganisms, the so-called “psychobiotics”, selected as a biotherapeutic tool for the maintenance of correct brain function through the microbiota–gut–brain axis [22,25].

Despite the promising potential of PA or probiotics for mitigating cognitive and brain deficits resulting from ageing and neurodegeneration, the mechanisms by which these strategies exert their effects in humans are not fully understood. In this review, we will discuss the effects of PA on improving cerebrovascular function, promoting adult neurogenesis, and reducing neuroinflammation as potential mechanisms linking exercise and brain health. Furthermore, we will describe the current evidence of the beneficial effects of probiotics on brain health, focusing on brain oxidative stress and the human clinical trials carried out evaluating the effects of different bacterial strains on mental health.

## 2. The Effect of Exercise on Cerebrovascular Function

Cerebrovascular function is an important determinant of brain health. Apart from age and genetic liabilities, the major risk factors for dementia and AD are of vascular nature [26,27]. Whilst the mechanisms linking cardiovascular disorder (CVD) risk factors and neurodegeneration are unclear, there is evidence to suggest that exercise-induced amelioration of CVD factors may be beneficial in the prevention of neurodegenerative disorders and counteracting cognitive aging.

### 2.1. Cerebral Blood Flow and Angiogenesis

Cerebral blood flow (CBF) supplies the brain with oxygen and nutrients it relies on to function properly. A reduction in CBF, also known as hypoperfusion, is a hallmark of brain ageing—with every decade of life, the CBF has been estimated to diminish by 5% [28]. Hypoperfusion has also been identified as a key pathological feature in neurodegenerative disorders and a major contributing factor in the development of mild cognitive impairment (MCI) and AD [29,30,31]. A lower CBF has been linked to a higher rate of future cognitive deficits in both non-demented elderly individuals and AD patients [30,32,33,34,35]. Notably, in healthy individuals, the association between age and CBF is influenced by cardiorespiratory fitness levels [36], with evidence suggesting that aerobic exercise can mitigate the effect of age on CBF and improve cognition in the healthy ageing population [37,38]. Similarly, engaging in a 12-month aerobic exercise programme was shown to improve CBF in older individuals with MCI, with the magnitude of improvement in the CBF marker directly correlated with improvements in memory function [39]. Whilst meta-analyses are currently lacking, the data from human studies linking aerobic exercise with improved CBF and cognitive performance is very encouraging. However, more long-term data is needed to determine if these beneficial effects persist and identify at which points the intervention is most efficacious.

A potential mechanism mediating the beneficial effects of exercise on cerebrovascular health and cognition is angiogenesis, i.e., the formation of new blood vessels. Exercise promotes the sprouting of new capillaries from pre-existing vessels, increasing capillary density [40]. This may be particularly beneficial in AD and PD, which are associated with a reduced density of microvessels in the brain [41,42]. Angiogenetic processes are regulated by vascular endothelial growth factors (VEGF)—a group of signalling molecules involved in the growth and maintenance of vascular and neuronal cells. Exercise promotes angiogenesis and increases the mRNA and protein levels of VEGF in both young and elderly individuals [43]. VEGF are produced primarily by myocytes and diffuse into the peripheral circuitry but have been shown to cross the blood–brain barrier (BBB) [44,45]. Therefore, it is likely that at least some of the positive effects of exercise are directly related to enhanced cerebral perfusion mediated by angiogenesis.

Indeed, a number of pre-clinical studies have demonstrated exercise-induced angiogenesis and provided evidence to support its cognitive-enhancing effects in-vivo. Prolonged PA—30 days of running on a wheel—was shown to promote angiogenesis in the motor cortex of rats as well as increase blood flow and volume as measured by functional magnetic resonance imaging [46]. Similarly, exercise-induced angiogenesis was reported in a mouse model of PD, with striatal (i.e., the brain region affected in PD) levels of angiogenetic markers VEGF and CD34 significantly increased following four weeks of treadmill training [47]. An increase in VEGF levels in response to exercise was also reported in aged rats [40]. Notably, in a TgCRND8 transgenic mouse model of AD amyloidosis, three months of running normalised hippocampal vascular morphology and preserved spatial memory [48]. Additionally, exercised-induced increase in muscle fibre VEGF levels was positively correlated with hippocampal-based memory and learning and VEGF levels in the hippocampus of rats [49]. Interestingly, VEGF have also been shown to directly enhance neurogenesis and synaptic function [50], which will be discussed in following sections.

### 2.2. BBB Integrity, Systemic Inflammation, and Clearance of Toxic Protein Aggregates

In the context of the prevention and slowing progression of PD and AD, it is important to note the effects of exercise on diminishing the permeability of BBB. The central nervous system (CNS) is separated from the peripheral tissues by BBB which regulates the entry of nutrients, molecules, and cells from the systemic circulation to the brain and vice-versa. It also plays a critical role in the clearance of cellular metabolites and toxins from the brain [51,52,53]. Disruption of BBB integrity (i.e., reduced barrier tightness and leakiness) occurs in ageing and neurodegeneration, leading to the infiltration of peripheral inflammatory mediators such as cytokines and macrophages and accumulation of toxic protein aggregates [53,54,55]. Loss of BBB integrity is associated with low-grade chronic inflammation, which affects a large proportion of the Western population [56,57] and has been implicated in the pathogenesis of neurodegenerative disorders [58]. In turn, the increase in inflammatory mediators entering the CNS following BBB dysregulation activates glial cells, triggering a second wave of inflammation and resulting in a vicious cycle of inflammation, leading to further damage of the BBB [26]. In this regard, exercise has been shown to exert neuroprotective effects by reducing systemic inflammation and therefore lowering the risk of infiltration into the CNS and improving the constitution of BBB tight junctions.

Regular PA has been reported to reduce the levels of pro-inflammatory cytokines, such as interleukin 6 (IL-6), tumour necrosis factor alpha (TNF-α), and monocyte chemoattractant protein-1 (MCP-1), which are associated with low-grade chronic inflammation [59,60,61,62], as well as increase the levels of anti-inflammatory cytokines [63]. Aranson and colleagues have demonstrated that in a group of healthy middle-aged individuals, the levels of C-reactive protein (CRP), a plasma biomarker of inflammation implicated in the pathogenesis of neurodegenerative disorders [64], decrease continuously with increased levels of physical fitness [65]. Furthermore, Chupel et al. have found combined taurine supplementation and exercise training to reduce levels of pro-inflammatory markers, improve BBB integrity, and increase cognitive performance in a group of elderly women [66]. Similar results have been seen in animal models—five weeks of running training improved the structural components of the BBB in diabetic rats, preserving the levels of claudin-5, a protein related to BBB integrity, and improving non-spatial memory [67]. Interestingly, exercise can also facilitate clearing of toxic metabolites such as amyloid beta (Aβ—the main component of amyloid plaques) by upregulating the expression of low-density lipoprotein receptor-related protein 1 (LRP-1) receptors on BBB, through which Aβ is transported out of the brain [68].

In conclusion, there is a large body of evidence to support the role of PA in maintaining cerebrovascular health. By increasing cerebral perfusion through angiogenesis, diminishing BBB permeability, reducing systemic inflammation, and aiding in the clearance of toxic protein aggregates, PA exerts neuroprotective effects that may counteract cognitive ageing and potentially prevent or delay the development of neurodegenerative disorders.

## 3. The Effect of Exercise on Neuroinflammation

**Figure 1:**
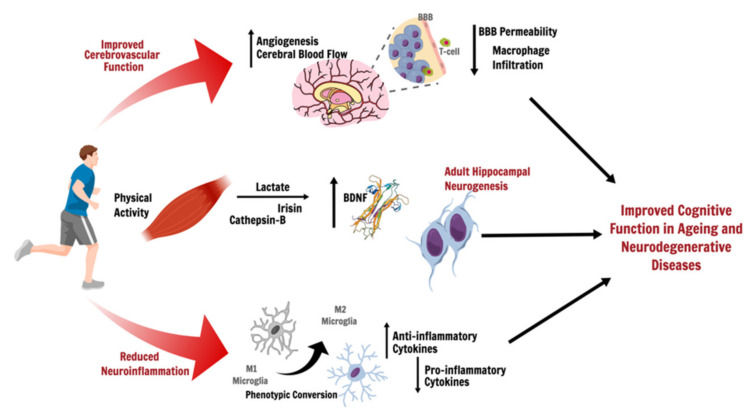
Mechanisms mediating the beneficial effects of exercise on brain health. Physical activity improves cognitive function in ageing and neurodegenerative diseases by improving cerebrovascular function, reducing neuroinflammation, and promoting adult hippocampal neurogenesis. Figure created using BioRender software.

In addition to the aforementioned effects of exercise on systemic inflammation, PA has also been shown to reduce neuroinflammation, which is a major feature of both AD/PD and ageing [69]. In the Tg2575 mouse model of AD, physically inactive mice were found to have a higher hippocampal expression of pro-inflammatory interleukins IL-1β and TNF-α, with reduced levels of interferon-gamma (IFN-γ) [70]. Three weeks of voluntary wheel running reduced the levels of IL-1β and TNF-α in the hippocampus and restored IFN-γ to wild-type levels, accompanied by a significant reduction in Aβ in the exercised group compared to sedentary animals [70]. PA has also been shown to mediate a phenotypic conversion of microglia, the primary immune cell in the CNS, from M1 (i.e., the classic “inflammatory” activation microglia sub-type) to M2 (i.e., the alternatively activated “neuroprotective” microglial sub-type) in a number of animal models [71,72,73] (see Figure 1). Jiang et al. [72] have shown that PA promoted the polarisation of microglia towards M2 phenotype and improved cognitive function in a rat model of chronic cerebral hypoperfusion. Similarly, in a streptozotocin (STZ)-induced rat model of AD, chronic treadmill exercise significantly inhibited reactive gliosis following STZ injection and shifted activated microglia from M1 phenotype to M2 [74]. This was accompanied by a reduction in pro-inflammatory markers and an upregulation of anti-inflammatory cytokine expression in the hippocampus of those animals as well as a significant preservation of hippocampal-dependent cognitive function [74]. Therefore, PA has the potential to modulate microglial phenotypes and promote anti-inflammatory effects in the brain, particularly the hippocampus, which at least in animal models appears to improve cognitive function.

## 4. The Effect of Exercise on Neurogenesis

Neurogenesis—the process of generating new functional neurons from neural stem and progenitor cells, is crucial for learning and memory function [75]. In the adult brain neurogenesis is largely restricted to two proliferative niches—the subventricular zone and the subgranular zone of the hippocampal dentate gyrus [76]. In animal models, ablation of hippocampal neurogenesis is associated with deficits in a range of hippocampus-dependent cognitive processes such as spatial memory, contextualised fear conditioning, and object recognition [77,78,79,80,81,82]. The rate of neurogenesis slows with age in both humans and laboratory animals [83,84,85,86,87], with impairments in the production of new neurons also reported in AD and PD as well as pre-clinical models of the diseases [88,89,90], thus suggesting that pro-neurogenic interventions may potentially mitigate cognitive deficits associated with ageing and neurodegeneration, which are likely to be translatable between pre-clinical models, and human conditions.

Exercise has been shown to promote neurogenesis in rodents with evidence suggesting the same may be true for humans [91,92,93]. Part of PA’s pro-neurogenic effects are thought to be due to its ability to reduce neuroinflammation, which is known to inhibit adult hippocampal neurogenesis (AHN) [94,95]. On the other hand, exercise-induced metabolic factors (i.e., lactate) and muscle-derived myokines (cathepsin-B and irisin) have been shown to stimulate the production of brain-derived neurotrophic factor (BDNF) [96,97,98,99,100,101,102], a growth factor involved in neuronal plasticity, synaptogenesis, and neurogenesis [103] (see Figure 1). In humans, the blood and brain levels of BDNF are reduced in AD and PD patients [104,105,106], with reports of a positive correlation between BDNF levels and cognitive performance [107,108]. Exercise is a potent stimulator of BDNF production—even a single session of intense PA has been shown to enhance the production of BDNF in both non-demented, healthy adults [109] and AD patients [109,110]. Similarly, a recent meta-analysis has demonstrated that PA interventions significantly elevate BDNF levels in PD patients, regardless of exercise type [111].

In rodents, wheel running is associated with an increase in mRNA and protein levels of BDNF within the hippocampus [92,112,113,114,115]. Furthermore, an elevation in hippocampal BDNF has been mechanistically linked to exercise-induced improvements in learning and memory mediated by the upregulation of signalling molecules cAMP response-element-binding protein (CREB) and synapsin I [116,117]. Indeed, inhibiting BDNF action in vivo blocks the beneficial effects of exercise on cognition—learning and memory function in exercised animals receiving BDNF blocker were reduced to sedentary control levels, highlighting the central role of BDNF in mediating exercise-induced cognitive benefits [118]. Notably, in a recent pre-clinical study, Choi and colleagues have found that inducing hippocampal neurogenesis via pharmacological or genetical means alone failed to improve cognitive function in a mouse model of AD [119]. In contrast, behavioural symptoms were ameliorated when hippocampal neurogenesis was induced by exercise and accompanied by an elevation in BDNF, interleukin 6 (IL-6), and synaptic markers. Interestingly, stimulating AHN in conjunction with the overexpression of BDNF mimicked exercised-induced improvements in cognition [119]. This suggests that promoting AHN can confer beneficial effects on cognition in AD, but only in the presence of a healthier brain environment optimal for the production of neurotrophins such as BDNF, which can be created through exercise, thus, further supporting the validity of PA as a non-invasive intervention with potent cognitive-enhancing effects. However, it is important to note that due to the inaccessibility of the human brain, most of what we know about the pro-neurogenic effects of exercise is drawn from animal models and indirect measures of human neurogenesis based on levels of BDNF and cognitive performance.

## 5. The Inhibition of Oxidative Stress through Exercise: The Influence of Different Exercise Type Programmes

**Figure 2:**
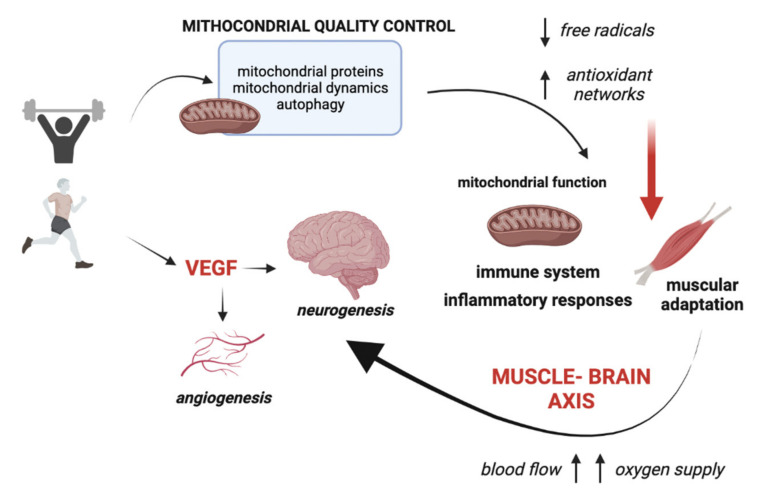
Mechanisms mediating the beneficial effects of specific training programmes on the muscle–brain axis. Resistance and aerobic training improves mitochondria function, immune system, and inflammatory response by improving mitochondria quality control, which leads to muscular adaptation, brain neurogenesis, and angiogenesis. Figure created using BioRender software.

Regular exercise is known to play an important preventive and therapeutic role in many diseases, as well as being beneficial to brain function [120,121,122]. Indeed, the increment of pumping and oxygenation of blood mediated by exercise creates an important environment to improve neuroprotective impact on brain function [123]. Moreover, previous studies have demonstrated that physical exercise (resistance and aerobic training) influence mitochondrial function and immune–inflammatory responses [124] through an important cellular process called mitochondria quality control [125]. This process includes the modification of mitochondrial proteins, mitochondrial dynamics, and autophagy [126], which leads to the control of the production of free radicals by creating antioxidant networks [127] (see Figure 2). As previously discussed, BDNF signalling through exercise plays a major role in neurogenesis, and the maintenance of cerebral BDNF levels is important for effective neural function [128]. However, it has been demonstrated that other neurotrophic factors such as vascular endothelial growth factor (VEGF) are involved in neurogenesis [129] (see Figure 2). In this line, Rich et al. [130] reported that exercise is an effective strategy to improve neurogenesis and angiogenesis in the hippocampus via VEGF production. However, it seems that the increased VEGF expression is transient after an acute bout of exercise (7–10 days), returning to baseline levels in a month [131]. Regardless, PA could be postulated as an important physiological stimulus capable of initiating protective brain mechanisms.

It is important to note that mitochondrial-induced ROS production during PA could have negative health consequences that are associated with oxidative stress in exercise practitioners [132]. In this line, the production of ROS is the result of the percentage of oxygen that the mitochondria has not been able to reduce during energy production [133]. However, it has been demonstrated that PA causes certain positive adaptations in the organism, such as the moderate elevations of ROS, which are involved in protective redox signalling and regulation [134]. Moreover, the elevation of these molecules by PA can regulate signalling or act as a signalling to muscular adaption [135] and produce adaptive responses to withstand further stress [136]. In addition, it has been shown that interindividual variability and training status can influence the levels of oxidative stress produced during exercise [137]. In this line, Margaritelis et al. [138] reported that different oxidative stress responses were observed in ninety-eight young men after five sets of eight eccentric maximal voluntary contractions, with an angular velocity of 60°/s with 2-minute rest intervals between sets. Thus, as a non-pharmacological strategy, PA has great potential to trigger regulatory responses that delay increased oxidative stress and decreased mitochondrial enzymatic activities [139].

This muscular adaptation and its reduction of oxidative stress shed light into the muscle–brain axis from a humeral point of view. In this line, a recent review [140] mentioned that a potential mechanism of PA benefits for the brain is the mitochondrial reprogramming, which is related to the increase of some systemic parameters (i.e., temperature, blood pressure, or oxygen supply, among others) in different tissues such as the muscle. Those parameters lead to the enhancement of clearance mechanisms of harmful substances, which reduce mitochondrial damage along the muscle–brain axis. In this line, Pereira et al. [141] have shown that increasing the blood flow in the dentate gyrus by cardiovascular training during 12 weeks improves the learning rate of an hippocampal-dependent task. Thus, an inadequate supply of oxygen would compromise mitochondrial function, negatively affecting brain function due to the increased oxidative stress [142].

Moreover, Erickson et al. [143] reported that PA training improves spatial memory function and reduces age-related hippocampal tissue loss in older adults. Furthermore, it is well established that PA is a potent stimulus to induce different signalling pathways that produce phenotypic changes in the mitochondria, leading to greater muscle health [126]. However, different methodological aspects of PA such as intensity and duration can exert different mitochondrial changes [140]. Thus, it has been shown that short-duration interval exercises with maximal and supramaximal intensities (high intensity interval training; HIIT or sprint interval training; or SIT) have a greater capacity to induce mitochondrial biogenesis than exercises performed at moderate intensity. Accordingly, MacInnis and Gibala [144] reported that 6–7 sessions of both HIIT and SIT produced 25–35% increasement of mitochondrial content. In addition, several studies have reported that high-intensity programmes elicited an increase in skeletal muscle mitochondrial respiration [145] and content [146] in comparison with moderate-intensity programmes. In conclusion, although endurance training was considered the primary means of achieving mitochondrial adaptation, performing alternative exercise training modalities could be an interesting strategy to induce similar improvements in mitochondria with a similar workload into a shorter period [133].

As previously described, oxidative stress can have different pathophysiological impacts [147]. Indeed, several studies have linked ROS’ reactivity to cellular damages such as lipoperoxidation, oxidation of protein sulfhydryl groups, and oxidation of purine and pyrimidine bases, which lead to the development of pathologies [148]. Indeed, the excessive production of ROS has been correlated to AD, vascular pathologies, and PD, among others [149].

Another complication of the increased ROS is the damage caused to mitochondria. ROS increasement has been proved to severely damage the mitochondria´s membranes, which leads to the reduction in the biogenesis of the mitochondria, which translates into a reduction in the production of ATP [150].

Exercise may increase the level, activation, and mRNA expression of endogenous antioxidant systems in the brain, and it has been shown to down-regulate the levels of the oxidative damage [151,152,153,154,155]. So, exercise has been implicated in reducing the risk of brain oxidative damage, but this response depends on the type of exercise used [155]. In this line, it has been shown that a single session of exhaustive exercise causes oxidative damage in untrained people. Indeed, the ROS released after a training session can cause damage at the lipid, protein, or DNA levels [156], whereas, in trained subjects these effects are not observed due to a greater resistance of these subjects to oxidative stress [157]. Additionally, harmful effects such as reduced force generation and increased muscle atrophy occur after non-regular strenuous exercise, whereas regular training has positive effects by influencing cellular processes leading to increased antioxidant expression [158]. These results proved evidence that different exercise interventions lead to different ROS responses.

### 5.1. The Relationship between Exercise and Increments of Oxidative Stress

Strong increases in ROS concentrations after strenuous exercise can cause contractile dysfunction and muscle atrophy, which promote muscle weakness and fatigue [159]. Other studies have shown that regardless of the type of acute exercise performed, aerobic [160] or anaerobic [161], there is an increase in ROS concentrations. ROS is naturally generated by the skeletal muscles when contracting; however, intense or prolonged exercise can result in increased oxidative damage production such as superoxide, hydrogen peroxide, and hydroxyl radicals.

Superoxide is formed primarily as an intermediate in many biochemical reactions. This anion is negatively charged and relatively impermeable to the membranes; however, compared to other free radicals, superoxide has a relatively long half-life, allowing its diffusion within the cell and, thus, increasing the number of potential targets to damage [162]. Regarding this, hydrogen peroxide (H_2_O_2_) is a reactive compound that leads to the generation of free radicals such as hydroxyl radicals. Hydrogen peroxide is stable, permeable to membranes with a relatively long half-life within the cell to which it is cytotoxic but with a relatively weak oxidizing agent [162]. Finally, hydroxyl radicals (OH^-^) are highly reactive with a strong oxidizing potential. Those features imply that when generated, they damage molecules near to their site of generation, although they are not permeable to membranes. The hydroxyl radicals are considered the most harmful ROS [162].

Several types of exercises have been linked to the production of the abovementioned ROS; we will shortly approach the most mentioned, such as resistance exercise and aerobic, anaerobic, and mixed exercises.

Resistance exercise has been linked to a rapid oxidative response after performing exercise [163,164], while others show hardly any effects [165]. Hudson et al. [166] have studied the relationship between acute moderate and high-intensity back squat exercise and the oxidative damage response as assessed by the biomarker protein carbonyls. Acute exercise has long been associated with a transient oxidative stress response [167]. The magnitude of oxidative stress following aerobic-type exercise is generally proportional to exercise intensity [160,168]. Additionally, evidence indicates that high-intensity resistance exercise, involving a large muscle mass such as squat exercise, consistently elicit a measurable blood oxidative stress [148,149,161,169]. Nonetheless, Hoffman et al. [137] demonstrated that plasma malondialdehyde values were correlated with an indirect marker of tissue oxygenation during the hypertrophy protocol as compared with the strength protocol. Those results are opposite to those of Hudson et al. [166] where the magnitude of carbonyl elevation was higher in the strength protocol than the hypertrophy protocol. Although both studies are supporting the positive relationship between intensity and oxidative stress, differences could be explained by the time course to complete and recover from the two protocols.

Regarding anaerobic exercise, an increasement in ROS production, lactic acid, acidosis, catecholamines, and post-exercise inflammation have been reported [170]. Precisely, this type of exercise significantly enhances purine catabolism and causes rapid deoxygenation named ischemia reperfusion phenomenon. These two phenomena are known to increase the activity of xanthine oxidase, which accelerates the production of ROS, more specifically O_2_^–^ and H_2_O_2_ [171].

Similar results linking intense exercise to the increase in ROS have been found in mice. Aguiar et al. [154] found that intense exercise promoted brain mitochondrial dysfunction as well as an increase in the frontal cortex thiobarbituric acid-reactive substance levels in exercised mice. In agreement to the above-mentioned study, Somani et al. [172] observed that different brain areas contained different activities of antioxidant enzymes, as well as glutathione peroxidise and oxidized glutathione (GPx and GSSG) levels, which were preferentially altered as a result of exercise training to cope with oxidative stress.

It is also important to note that exercise-induced oxidative stress has wide interindividual variability [163]. Kawamura and Muraoka [173] studied 98 subjects performing eccentric quadriceps exercise and red blood cell, plasma, and urine samples were collected immediately after exercise and two days post-exercise. In the study, three biomarkers related to oxidative stress were analysed, such as F2-isoprostanes, protein carbonyls and glutathione. A considerable number of the participants exhibited changes in biomarker levels in the opposite direction to the group average. Indeed, 13% of the participants exhibited a decrease in F2-isoprostanes and protein carbonyls, whereas 10% of the participants showed an increase in glutathione levels. Furthermore, one out of three individuals showed unexpected or negligible responses (0% to ± 5%) to exercise in at least one redox biomarker [164]. These data highlighted the importance of inter-individual variability. This variability could be influenced by the training levels of the subjects, their level of rest, and the intake of antioxidants or even psychological variables, all of which should be taken into account when performing these studies.

### 5.2. Exercise as a Strategy to Reduce Oxidative Stress

Moderate aerobic training or simply voluntary exercise, such as running on a wheel, ameliorates antioxidant capacity [174,175,176,177,178,179,180], and regular moderate exercise improves brain function [177], memory [176], proteasome activation, and up-regulation of the antioxidant system [181]. Furthermore, daily moderate exercise has been shown to reduce damage of hippocampal slices from Wistar rats exposed to in vitro ischemia [182,183]. As previously described, anaerobic exercise in a progressive exercise programme can also improve different activities of antioxidant enzymes in the brain [172]. Similarly, anaerobic exercise with 10 s (short) or 40 s (long) rest intervals increased the antioxidant capacity from different tissues [184] at the same time that running on a treadmill until exhaustion did not induce lipid peroxidation by oxidative stress in the hippocampus [185]. Surprisingly, some other studies in which rats were overtrained in long terms of strenuous exercise or when the duration increased abruptly did not induce brain oxidative stress [186,187,188], and neither did similarly acute and chronic exercise promote oxidant stress in the prefrontal cortex, striatum, and hippocampus [189]. Those results are opposite to the abovementioned studies, where aerobic extenuation or anaerobic programs have been found to increase oxidative stress, pointing out the need to further investigate the key factors attenuating those oxidative effects.

The literature supporting a positive relationship between aerobic exercise and the reduction of oxidative stress is much clearer [167,190]. It has been shown that aerobic exercise leads to an increase in maximal oxygen consumption (VO_2_max) and an increase in ROS production [168]. However, if aerobic exercise intensities do not exceed 50% of VO_2_max, ROS production is reduced to minimal values, as demonstrated in studies carried out by Ashton et al. [191] or by Chevion et al. [192]. Additionally, aerobic exercise has been shown to promote a positive effect on SOD levels in 100% of the cases and improved lipid peroxidation in 90% of the studies [176,178,179,180,183,188,189,193].

In conclusion, aerobic exhausted exercise, anaerobic exercise, or the combination of both types of training still report confusing findings in relation to the production of oxidative stress. In contrast, regular moderate aerobic exercise appears to be highly contrasted to protect against brain oxidative stress. More research into factors causing inter-individual variability when performing each type of training would shed light into the relationship between exercise and oxidative stress.

## 6. The Beneficial Effects of Probiotics on Brain Health

**Figure 3:**
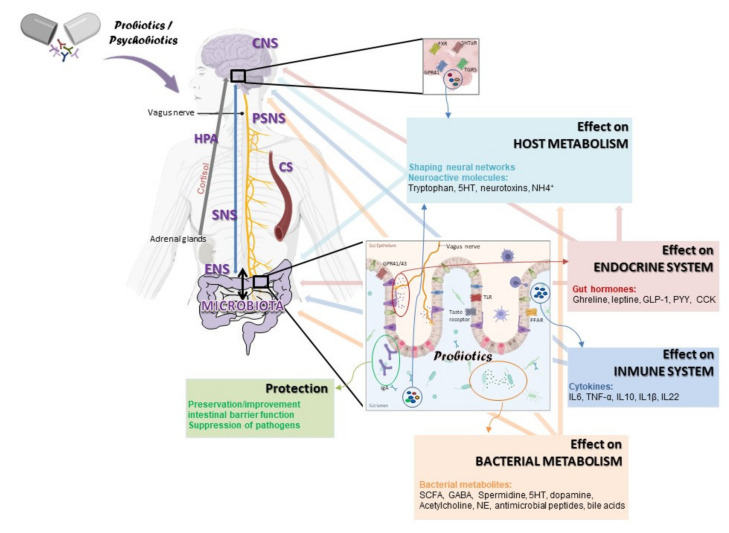
Routes of communications between gut microbiota and brain and mechanisms through probiotics keep homeostasis between both systems. CNS: central nervous system; SNS: sympathetic nervous system; PSNS: parasympathetic nervous system; ENS: enteric nervous system; HPA: hypothalamic-pituitary-adrenal; CS: circulatory system; GABA: α-aminobutyric acid; SCFA: Short chain fatty acids; 5HT: serotonin; NE: norepinephrine; NH4+: ammonium; GLP-1: glucagon-like peptide -1; PYY: peptide YY; CCK: cholecystokinin; IL: interleukin; TNF-α: tumor necrosis factor α; TLR: toll-like receptors; FXR: bile acid farnesoid X receptor; TGR5: bile acid Takeda G-protein-coupled receptor 5; GPR41/43: G protein-coupled receptors 41/43; 5HTa R: serotonin receptor. Figure created using BioRender software.

The central nervous system is functionally communicated with the gastrointestinal tract. While brain signals different from motor, sensory, and secretory functions have an impact on the gastrointestinal system, signals from the gut also influence brain function [194,195]. This bidirectional communication, known as the “gut-brain axis”, includes the conjunction of the central nervous system, the enteric nervous system, the parasympathetic and sympathetic nervous systems, the endocrine-immune system, and the hypothalamus-pituitary-adrenal axis, in addition to the circulatory system [22]. Over the past few years, the gut microbiota has emerged as another key player in the interaction between gut and brain [196,197], leading to the coining of the phrase: “the microbiota-gut-brain axis”. The gut microbiota is a complex and dynamic community of microorganisms including bacteria, viruses, protozoa, fungi, and archaea that has co-evolved with the carrier host [198]. A total of 1014 microorganisms was reported in the gut, comprising 150 times more genes than the total number of human genes [107], which is called the “microbiome”. This “forgotten organ” [199] exerts essential functions for the host with effects beyond the gastrointestinal environment and is a milestone for physiological homeostasis maintenance [14]. Among those functions, intestinal microbiota trains the host immune system, participates actively in the gut barrier integrity and metabolism, and produces and controls the production of vitamins, hormones, and neurotransmitters [200,201]. Recently, it has been demonstrated that gut microbiota impact hypothalamus and amygdala functions [202,203] that are specially implicated in stress [204]. Evidence continues to demonstrate that the gut microbiota is particularly implicated in brain physiology and behaviour, affecting host mental health [17,18,22]. The dynamic nature of the gut microbiota makes it highly responsive to external factors. It is powerfully demonstrated that the age of the subject, the dietary pattern, exercise habits, medications, or stress affect the microbiota composition [205,206]. The imbalance of the gut microbiota, a status known as “dysbiosis” (despite to be not known yet the exactly definition of this microbiota alteration), can involve an altered signalling from the gut to brain, negatively influencing brain health or vice versa [22] (see Figure 3).

Accordingly, the gut microbiota is being increasingly used as a key target for both dietary and therapeutic strategies interventions to finally modulate the gut-brain axis. One promising approach entails the use of probiotics, which are defined as “live microorganisms that, when administered in adequate amounts, confer a health benefit on the host” [207]. Last two decades’ research have evidenced the impact of probiotics on gut microbiota physiology, establishing a clear strain-specificity and population- or diseases-specificity and knocking over the traditional concept of the “golden-strain” [208]. In recent years, different and specific probiotics strains have emerged by their beneficial effects in particular stages of life or diseases [16,208,209]. With respect to mechanisms of action, they have been described in different ways by which probiotics work, including the enhancement of the epithelial barrier, increased adhesion to intestinal mucosa and concomitant inhibition of pathogen adhesion, bacteriocins and acids production, the inhibition of bacterial translocation, anti-inflammatory substances production and immune system modulation, vitamins and neurotransmitters production, effect on calcium-dependent potassium channels in intestinal sensory neurons, and the induction of opioid and cannabinoid receptors in intestinal epithelial cells, among others [209,210] (see Figure 3).

Taken this together, it is clear that certain probiotics strains can modulate various features of the microbiota–gut–brain axis affecting positively on brain health. Most of the research was conducted in pre-clinical models (see Table 1), but there is also a significant body of evidence resulting from human studies. The reported beneficial findings of psychobiotics range from effects on physiological stress to anxiety, depression, mood, pain, or cognition [22]. In the Table 2, the majority of the clinical studies with probiotic strains showing effects on brain health have been compiled. Most of these studies did not set out to test the specific biological mechanisms that could be underlying the positive behavioural or mental health. However, as it was stated before, there is extended literature showing different hypotheses. Some biomarkers of inflammation and oxidative stress including interleukin 6, tumour necrosis factor alpha, catalase, or superoxide dismutase (SOD) could be of interest and very informative about this yet unknown process. Indeed, in two clinical trials with AD´s patients examining different probiotics combinations [211,212], an improvement in the Mini-mental state examination score were observed together with a significant increase in total antioxidant capacity and total glutathione (GSH) and a significant reduction in high sensitivity C-reactive protein. It is also worthy to mention that not all the probiotics tested in clinical trials entailed positive effects, even using the same strain or combination of strains in different populations. For example, the combination of the strains *Lactobacillus helveticus* R0052 and *Bifidobacterium longum* R0175 showed positive effects on anxiety and depression parameters in three different studies [213,214,215]; however, Romjin et al. [216] did not showed any evidence that the same probiotic formulation was effective in treating low mood. *Limosilactobacillus reuteri* DSM 17938 (former *Lactobacillus reuteri* DSM 17938) showed effectiveness in the alleviation of pain in a cohort of children aged at 4–18 years with functional abdominal pain and inflammatory bowel disease [217]; however, no effects on neurocognitive and sensory outcomes were observed in a cohort of very low birth-weight preterm infants [218].

**Table 1:** Effects of exercise on brain health in pre-clinical studies.

Study	Type of Exercise	Type of Neurodegenerative	Effects on Brain Health
**Pre-Clinical**			
Ding et al. [129]	Aerobic exercise	Healthy	Angiogenesis
Swain et al. [46]	Aerobic exercise	Healthy	Angiogenesis in the motor cortex Increase blood flow and volume
Maliszewska-Cyna et al. [48]	Aerobic exercise	AD	Normalise hippocampal vascular morphology and preserved spatial memory
Karakilic et al. [49]	Aerobic exercise	Healthy	Increase in muscle fibre VEGF levels was positively correlated with hippocampal-based memory and learning, and VEGF levels in the hippocampus
De Senna et al. [67]	Aerobic exercise	Diabetic Rats	Improves Non-Spatial Memory, Locomotor Skills, and the BBB
Herring et al. [68]	Aerobic exercise	AD	Facilitate clearing of toxic metabolites such as amyloid beta (Aβ) by upregulating the expression of low-density lipoprotein receptor-related protein 1 (LRP-1) receptors on BBB through which Aβ is transported out of the brain
Nichol et al. [70]	Aerobic exercise	AD	Reduced the levels of IL-1β and TNF-α in the hippocampus and restored IFN-γ to wild-type levels, accompanied by a significant reduction in Aβ
He et al. [71]	Aerobic exercise	Healthy	Promotes Glymphatic Clearance of Aβ and Reduces the Activation of Astrocytes and Microglia
Jiang et al. [72]	Aerobic exercise	Healthy	Improves cognitive function together with microglia phenotype modulation and remyelination in chronic cerebral hypoperfusion
Kohman et al. [73]	Aerobic exercise	Healthy	Reduces activation of microglia isolated from hippocampus and brain
Lu et al. [74]	Aerobic exercise	AD	Inhibite reactive gliosis following STZ injection and shifted activated microglia from M1 phenotype to M2. Preservation of hippocampal-dependent cognitive function
Van Praag et al. [91]	Aerobic exercise	Healthy	Increases cell proliferation and neurogenesis
Liu and Nusslock [92]	Aerobic exercise	Healthy	Mediated neurogenesis in the hippocampus via BDNF
Moon et al. [96]	Aerobic exercise	Healthy	Induce systemic Cathepsin B secretion is associated with memory function
Wrann et al. [97]	Aerobic exercise	Healthy	Exercise induces hippocampal BDNF through a PGC-1α/FNDC5 pathway
Lourenco et al. [98]	Aerobic exercise	AD	Increase hippocampal FNDC5/irisin in patients at risk of developing AD or in patients already exhibiting cognitive impairment
Hayek et al. [99]	Aerobic exercise	Healthy	Exercise induces the Mus musculus Bdnf gene and promotes learning and memory formation
Oliff et al. [112]	Aerobic exercise	Healthy	Increase in mRNA and protein levels of BDNF within the hippocampus
Van Hoomissen et al. [113]			
Adlard et al. [114]			
Lee and Soya [115]			
Vaynman et al. [116]	Aerobic exercise	Healthy	Exercise induces improvements in learning and memory mediated by upregulation of signalling molecules cAMP response-element-binding protein (CREB) and synapsin I
Choi et al. [119]	Aerobic exercise	AD	Behavioural symptoms were ameliorated when hippocampal neurogenesis was induced by exercise and accompanied by elevation in BDNF, interleukin 6 (IL-6) and synaptic markers
Cotman and Engesser [128]	Aerobic exercise	Healthy	Exercise increases in brain-derived neurotrophic factor, a molecule that increases neuronal survival, enhances learning and protects against cognitive decline
Rich et al. [130]	Aerobic exercise	Healthy	Exercise is an effective strategy to improve neurogenesis and angiogenesis in the hippocampus via VEGF production
Pereira et al. [141]	Aerobic exercise	Healthy	Improves the learning rate of an hippocampal-dependent task Reduce mitochondrial damage along the muscle–brain axis
Um et al. [151]	Aerobic exercise	AD	Exercise may increase the level, activation, and mRNA expression of endogenous antioxidant systems in the brain, and it has been shown to down-regulate the levels of the oxidative damage
Aguiar et al. [152]	Aerobic exercise (downhill training)	Healthy	Downhill running is as effective as level running in increasing hippocampal BDNF protein levels; BDNF protein is elevated in the striatum after downhill physical training.
Aguiar et al. [153]	High-Intensity Physical Exercise	Healthy	Cellular signalling disturbances were associated with poor antioxidant response in the basal ganglia and with implicit memory impairment.
Aguiar et al. [154]	Short bouts of mild-intensity physical exercise	Healthy	Improvement of age- related spatial memory déficitsIncreased hippocampal plasticity via AKT, CREB, and BDNF signalling
Tuon et al. [155]	Aerobic exercise	PD	Protective effect on PD-induced 6-OHDA, possibly due the ability of exercise to modulate the brain redox state and preserve the content of the proteins that are important for normal brain function
Somani et al. [172]	Aerobic exercise	Healthy	Exercise training causes more oxidative stress in the brainstem (BS) and corpus striatum (CS) regions, or has a better ability to induce antioxidant enzymes to cope with the superoxides formed. BS and CS may be more sensitive to oxidative stress
Somani et al. [172]	Anaerobic exercise	Healthy	Improve different activities of antioxidant enzymes in brain
Radák et al. [157]	Aerobic exercise	Healthy	Improve memory function
Radák et al. [177]	Aerobic exercise	Healthy	Improve brain function
Radák et al. [181]	Aerobic exercise	Healthy	Improve proteasome activation, and up-regulation of the antioxidant system
Scopel et al. [182]	Aerobic exercise	Healthy	Reduce damage of hippocampal rats exposed to in vitro ischemia
Cechetti et al. [183]			
Acikgoz et al. [185]	Anaerobic exercise	Healthy	Running on a treadmill until exhaustion did not induce lipid peroxidation by oxidative stress in the hippocampus
Fry et al. [186]	Moderate to strenous exercise	Healthy	Not induce brain oxidative stress
Petibois et al. [187]	Moderate to strenous exercise	Healthy	Not induce brain oxidative stress
Ogonovszky et al. [188]	Moderate to strenous exercise	Healthy	Not induce brain oxidative stress
Aksu et al. [189]	Acute and chronic exercise	Healthy	Acute and chronic exercise neither promoted oxidant stress in prefrontal cortex, striatum, and hippocampus

AD, Alzheimer’s disease; PD, Parkinson.

**Table 2:** Effects of exercise on brain health in clinical studies.

Study	Type of Exercise	Type of Neurodegenerative	Effects on Brain Health
**Clinical**			
Santos-Lozano et al. [9]	≥150 min/week of moderate-intense activity	AD	40% reduction of risk for development of AD
Zimmerman et al. [36]	Aerobic exercise	Healthy	Mitigate the effect of age on CBF
Ainslie et al. [37]			
Lucas et al. [38]			
Thomas et al. [39]	Aerobic exercise	MCI	Mitigate the effect of age on CBF Improvements in memory function
Gavin et al. [43]	Aerobic exercise	Healthy	Increases the mRNA and protein levels of VEGF
Aronson et al. [65]	Different levels of PA	Healthy	Levels of CRP decrease continuously with increased levels of physical fitness
Chupel et al. [66]	Combined exercise training programme (resistance training and aerobic exercise)	Healthy	Reduce levels of pro-inflammatory markers, improve BBB integrity, and increase cognitive performance
Coelho et al. [109]	Aerobic exercise	Healthy	Enhance the production of BDNF
Kwak [110]	Aerobic exercise	AD	Improvements on BDNF Peripheral Levels and Cognition
Ruiz-González et al. [111]	Aerobic exercise	PD	Elevate BDNF levels
Rashid et al. [123]	Aerobic exercise	AD	Neuroprotective impact on brain function Increase in angiogenesis, neurogenesis, and synaptogenesis mainly due to an increase in blood flow, brain-derived neurotrophic factor (BDNF), insulin-like growth factor 1 (IGF-1), hormones, and second messengers.
Murphy et al. [142]	Different types of exercise	Healthy	Exercise improves spatial memory function and reduced age-related hippocampal tissue loss in older adults

AD, Alzheimer’s disease; PD, Parkinson’s disease; MCI, Mild cognitive impairment.

In the gut-brain axis context, a new definition for probiotics was raised. “Live organism that, when ingested in adequate amounts, produces a health benefit in patients suffering from psychiatric illness” was coined as “phychobiotics” by Dinan et al. [25]. This definition was later expanded to include prebiotics and also human populations at risk [219]. Nowadays, defining the action mode of psychobiotics at a cellular level, and how it mediates beneficial effects at a mechanistic level, is a challenge and hot topic field for neuroscientists and microbiologists, and it is a multidisciplinary field with high potential health benefits. To date, different ways or mechanisms through which probiotics can affect the gut-brain axis have been proposed (Figure 3). It is known that certain probiotics strains can produce neurochemicals, such as gamma-aminobutyric (GABA) [220] or acetylcholine [221], important neurotransmitters in the human brain. Intestinal bacterial was also observed to be involved in the production or metabolism of serotonin, norepinephrine, or dopamine [25]. Thus, a huge range of neurotransmitters can be produced by gut bacteria, being some of them key players in gastrointestinal and brain health. Moreover, inflammatory mediators such as cytokines or hormones such as cortisol, which can be modulated by intestinal bacteria or probiotics, are involved in this bidirectional communication [222]. In vivo studies have shown how the ingestion of certain probiotic strains decreased the levels of corticosterone and pro-inflammatory cytokines, with brain health benefits for the host [22]. On the other hand, some probiotics or psychobiotic strains produce short chain fatty acids (SCFA), which can cross the blood–brain barrier and exert neuroprotective and antidepressant properties [223], but it was also observed how SCFA reverted morphology deficits in microglia cells and enhanced stress levels in mice [22]. In a key study, Bravo et al. [210] demonstrated another communication path between the gut and brain, the vagus nerve, after vagotomy experiments with a probiotic strain (see Table 3).

It is also worthy to mention that only a limited range of probiotics have been tested as psychobiotics, with strains belonged to the genus *Bifidobacterium* and *Lactobacillus* being the most used [219], and most of them were administered in different mixes or combinations. Moreover, most of those strains were not selected based on their specific properties for gut-brain axis modulation but by their commercial availability or by other physiological beneficial effects. Accordingly, future studies are needed to select specific and targeted psychobiotic strains intended to specific disorders, and the use of one strain should not be generalized to another destination without its validation by independent studies. Finally, studies focusing on the mechanisms underlying the beneficial mental health action of the probiotics are required.

**Table 3:** Studies evaluating the effects of probiotics on brain health.

Probiotic Used	Human Cohort	Beneficial Effects	Reference
**Anxiety and depression**			
*Lactobacillus casei* strain Shirota	Chronic fatigue syndrome patients	Decreased anxiety symptoms	Rao et al. [224]
*Lactobacillus helveticus* R0052, *Bifidobacterium longum* R0175	Healthy adult volunteers	Alleviated psychological distress. Better anxiety, depression, anger-hostility, and problem-solving parameters	Messaoudi et al. [213]
*L. helveticus* R0052, *B. longum* R0175	Adults with UFC <50 ng/ml at baseline	Alleviated psychological distress. Decreased anxiety, depression parameters	Messaoudi et al. [214]
*Lactobacillus acidophilus* CUL60 (NCIMB 30157), *L. acidophilus* CUL21 (NCIMB 30156), *Bifidobacterium animalis ssp. lactis* (*B. lactis*) CUL34 (NCIMB 30172), *Bifidobacterium bifidum* CUL20 (NCIMB 30153)	Healthy adult volunteers	Decreased anxiety scores	Owen et al. [225]
*L. acidophilus, L. casei, B. bifidum*	Adult patients with major depressive disorder	Decreased Beck´s Depression Inventory total scores	Akkasheh et al. [226]
Probiotic A*: L. acidophilus* LA5, *B. lactis* BB12. Probiotic B: *L. casei*, *L. acidophilus*, *Lactobacillus rhamnosus*, *Lactobacillus delbrueckii* ssp. *bulgaricus (L. bulgaricus)*, *Bifidobacterium breve, B. longum*, *Streptococcus thermophilus* plus fructo-oligosaccharides	Healthy adult volunteers	Improvement in depression and anxiety scores	Mohammadi et al. [227]
*L. casei* strain Shirota	Healthy adult volunteers	Prevention of cortisol hyper-secretion and physical symptoms under stressful conditions	Takada et al. [228]
*L. acidophilus*, *B. bifidum*, *S. thermophilus*	Resistant depression adult patients being currently depressed	Decreased depression scores	Bambling et al. [229]
*S. thermophilus* (CNCM I-1630), *L. bulgaricus* (CNCM I-1632, I-1519), *Lactococcus lactis* ssp. *lactis* (CNCM I-1631), *L. acidophilus*, *S. thermophilus*, *Lactobacillus plantarum*, *B. lactis* (CNCM I-2494), *Lactobacillus reuteri* (DSM 17938) (in combination with maltodextrin, silica, casein, lactose and gluten)	Healthy adult volunteers	Decreased Hamilton´s anxiety scores	Colica et al. [230]
*B. longum* NCC3001	Patients with IBS and diarrhoea	Decreased depression scores	Pinto-Sanchez et al. [231]
*L. rhamnosus* HN001	Pregnant women	Lower depression and anxiety scores in postpartum period	Slykerman et al. [232]
*L. casei* W56*, L. acidophilus* W22*, Lactobacillus paracasei* W20*, B. lactis* W51*, Lactobacillus salivarius* W24*, Lactococcus lactis* W19*, B. lactis* W52*, L. plantarum* W62 *and B. bifidum* W23	Healthy adult volunteers	Improvement in depression and anxiety scores	Bagga et al. [233]
*L. casei, L acidophilus, L bulgarigus, L rhamnosus, B. breve, B. longum, S. thermophilus* (in combination with antidepressants and fructooligosaccharides)	Moderate depression adult patients	Decreased Hamilton rating scale for depression	Ghorbani et al. [234]
*L. helveticus* Rosell-52 (R005), *B. longum* Rosell-175 (R0175)	Adult patients with major depressive disorder	Improvement in depression scores	Kazemi et al. [215]
*Bacillus coagulans* MTCC 5856	Adult IBS patients with major depressive disorder	Decreased depression scores	Majeed et al. [235]
*Clostridium butyricum* MIYAIRI 588 (CBM588) (in combination with antidepressants)	Treatment-resistant major depressive disorder patients	Improvement in depression scores	Miyaoka et al. [236]
*B. breve* strain A-1	Schizophrenia patients	Improvement in depression and anxiety scores	Okubo et al. [237]
*L. plantarum* P8	Stressed adults	Reduced anxiety scores	Lew et al. [238]
*B. bifidum* W23, *B. lactis* W51, *B. lactis* W52*, L. acidophilus* W37, *Lactobacillus brevis* W63, *L. casei* W56, *L. salivarius* W24, *Lactococcus lactis* W19, *Lactococcus lactis* W58	Female health care workers employed on a rotating-shift schedule	Improvement in anxiety and fatigue	Smith-Ryan et al. [239]
**Mood**			
*L. casei* strain Shirota in milk drink	Healthy adult volunteers	Mood improvement	Benton et al. [240]
*B. animalis* ssp *lactis* (CNCM I-2494), *S. thermophilus* (CNCM I-1630), L*. bulgaricus* CNCM I-1632, *L. bulgaricus* I-1519, *Lactococcus lactis* ssp *lactis* (CNCM I-1631) in fermented milk	Healthy women volunteers	Changes in activity of brain regions controlling emotion and sensation	Tillisch et al. [241]
*B. bifidum* W23, *B. lactis* W52, *L. acidophilus* W37, *L. brevis* W63, *L. casei* W56, *L. salivarius* W24, *Lactococcus lactis* W19, *Lactococcus lactis* W58	Healthy adult volunteers	Reduced cognitive reactivity to sad mood (rumination and aggressive thoughts)	Steenbergen et al. [242]
*Lactobacillus fermentum* LF16, *L. rhamnosus* LR06, *L. plantarum* LP01, *B. longum* BL04	Healthy adult volunteers	Improvement depressive mood state, anger, fatigue, and sleep quality	Marotta et al. [243]
**Stress**			
*L. acidophilus* Rosell-52, *B. longum* Rosell-175	Stressed adults volunteers	Reduced two stress-induced gastrointestinal symptoms (abdominal pain and nausea/vomiting)	Diop et al. [244]
*B.longum* 1714	Healthy adult volunteers	Reduced daily reported stress	Allen et al. [245]
*L. casei* strain Shirota	Healthy adult students volunteers	Decreased stress-associated responses of abdominal dysfunction measured by feelings of stress and salivary cortisol levels	Kato-Kataoka et al. [246]
Probiotic A: *L. acidophilus* LA5, *B. lactis* BB12. Probiotic B: *L. casei*, *L. acidophilus, L. rhamnosus, L. bulgaricus, B. breve, B. longum, S. thermophilus* (in combination with fructo-oligosaccharides)	Healthy adult volunteers	Improvement in stress scores	Mohammadi et al. [227]
*L. plantarum* DR7	Stressed adults volunteers	Reduced symptoms of stress and anxiety	Chong et al. [247]
*L. plantarum* P8	Stressed adults volunteers	Reduced stress scores	Lew et al. [238]
**Cognition**			
*L. acidophilus* CUL60 (NCIMB 30157), *L. acidophilus* CUL21 (NCIMB 30156), *B. lactis* CUL34 (NCIMB 30172), *B. bifidum* CUL20 (NCIMB 30153)	Healthy adult volunteers	Improvement in attention tasks	Owen et al. [225]
*L. acidophilus, L. casei, B. bifidum, L. fermentum* in milk drink	Alzheimer’s disease patients	Improed performance in the Mini-mental state examination score	Akbari et al. [211]
*B*. *longum* 1714	Healthy adult volunteers	Improved hippocampal-dependent visuospatial memory performance and enhanced frontal midline electroencephalographic mobility	Allen et al. [245]
*L. plantarum* DSM 24730, *S. thermophilus* DSM 24731, *B. breve* DSM 24732, *L. paracasei* DSM 24733, *L. delbrueckii* ssp. *bulgaricus* DSM 24734, *L. acidophilus* DSM 24735, *B. longum* DSM 24736, *Bifidobacterium longum* ssp. *infantis* (*B. infantis*) DSM 24737	HIV-1 infected patients	Improvement in several neurocognitive tests	Ceccarelli et al. [248]
*L. casei* W56*, L. acidophilus* W22*, Lactobacillus paracasei* W20*, B. lactis* W51*, Lactobacillus salivarius* W24*, Lactococcus lactis* W19*, B. lactis* W52*, L. plantarum* W62*, B. bifidum* W23	Healthy adult volunteers	Improvement in emotional memory and emotional decision-making tasks	Bagga et al. [233]
*L. casei* W56, *L. acidophilus* W22, *L. paracasei* W20, *B. lactis* W51, *L. salivarius* W24, *Lactococcus lactis* W19, *B. lactis* W52, *L. plantarum* W62, *B. bifidum* W23	Healthy adult volunteers	Behaviour modulation and a shift towards efficient attentional control	Bagga et al. [249]
*B. bifidum* W23, *B. lactis* W51, *B. lactis* W52, *L. acidophilus* W37, *L. brevis* W63, *L. casei* W56, *L. salivarius* W24, *L. lactis* W19, *Lactococcus* lactis W58	Adult patients with mild to severe depression	Reduced cognitive reactivity towards sad mood	Chahwan et al. [250]
*L. plantarum* DR7	Stressed adults volunteers	Improved cognitive and memory functions in adults >30 years old	Chong et al. [247]
*L. helveticus* IDCC380	Healthy older volunteers	Improvement in the performance of cognitive tests	Chung et al. [251]
*B. breve* A1	Older adults with mild cognitive impairment or memory complaints	Improvement in several neurocognitive tests	Kobayashi et al. [252,253]
*L. plantarum* P8	Stressed adults	Improvement in memory and cognitive traits (social emotional cognition, verbal learning, and memory upon application)	Lew et al. [238]
*Lactobacillus gasseri* CP2305	Healthy young adults exposed to chronic stress	Reduced anxiety and sleep disturbance	Nishida et al. [254]
*B. bifidum* W23, *B. lactis* W51, *B. lactis* W52, *L. acidophilus* W37, *L. brevis* W63, *L. casei* W56, *L. salivarius* W24, *Lactococcus lactis* W19, *Lactococcus lactis* W58	Healthy women volunteers	Cognitive improvement under induced acute stress	Papalini et al. [255]
*L. plantarum* 299v (in combination with antidepressants)	Adult patients with major depressive disorder	Improvement in cognitive performance (attention, perceptivity, and verbal learning)	Rudzki et al. [256]
*L. acidophilus, B. bifidum, B. longum* (in combination with selenium)	Alzheimer’s disease patients	Improvement Mini-mental state examination score	Tamtaji et al. [212]
*B. longum* 1714	Healthy adult volunteers	Reduced mental fatigue and modulated neural responses during social stress	Wang et al. [257]
**Pain**			
*B. infantis* M-63, *B. breve* M-16V, *B. longum* BB536	Children with IBS (age 8–17.9 years) and children with FD (age 8–16.6 years)	Improvement in abdominal pain and frequency in IBS children	Giannetti et al. [258]
*L. reuteri* DSM 17938	Children (age 4-18 years) with treatment of functional abdominal pain and IBS in children.	Reduced intensity and duration of pain	Jadrešin et al. [217]
**ASD**			
*L. acidophilus* (strain Rosell-11)	Children with autism (age 4–10 years)	Improvement in the ability of concentration and carrying out orders	Kałużna-Czaplińska et al. [259]
*L. acidophilus*, *L. case*, *Lactobacillus delbruecki*, *B. longum, B. bifidum* formulated with the immunomodulator Del-Immune V® (*L. rhamnosus* V lysate)	Children with autism	Improvement all autism treatment evaluation checklist domains (speech, language, communication, sociability, sensory, cognitive awareness, and health, physical, and behaviour)	West et al. [260]
*L. acidophilus*, *L. rhamnosus*, *B. longum*	Children with autism (age 5–9 years)	Improvements in the severity of autism	Shaaban et al. [261]
*L. plantarum* PS128	Children with autism (age 7–15 years)	Ameliorated opposition/defiance behaviours total score. Improvement in attention deficit hyperactivity disorder and oppositional defiant disorder in younger children (aged 7–12 years)	Liu et al. [262]

UFC: urinary free cortisol; IBS: irritable bowel syndrome; HIV-1: immunodeficiency virus-1; FD: functional dyspepsia; ASD: autism syndrome disorder. Taxonomy from *Lactobacillus* genus was recently revised (Zheng et al. 2020) and the former species: *Lactobacillus brevis, Lactobacillus casei, Lactobacillus fermentum, Lactobacillus paracasei, Lactobacillus plantarum, Lactobacillus reuteri, Lactobacillus rhamnosus* and *Lactobacillus salivarius* are now referred to as *Secundilactobacillus collinoides, Lacticaseibacillus casei, Limosilactobacillus fermentum, Lacticaseibacillus paracasei, Lactiplantibacillus plantarum, Limosilactobacillus reuteri, Lacticaseibacillus rhamnosus* and *Ligilactobacillus salivarius,* respectively.

## 7. Probiotics as a Therapeutic Tool against Brain Oxidative Stress

As previously stated, oxidative stress and the associated damage constitute a common trait in different disorders, and it is associated with ageing. Therefore, maintaining the redox balance results are critical for host homeostasis in general and for brain function in particular. This has attracted the attention of the research community towards the development of intervention strategies aimed at reducing this oxidative stress as a key step for health maintenance. The use of dietary antioxidant compounds has been the most studied approach, but others have also been evaluated. To this regard, although not one of the most commonly explored mechanisms, the use of probiotics for their antioxidative properties has been assessed.

The seminal studies in this area were performed with microorganisms of the genus Lactobacillus more than two decades ago by selecting strains well suited to deal with reactive oxygen species [263]. A strain selected in these early studies, *Limosilactobacillus fermentum* (formerly *Lactobacillus fermentum*) strain ME3 (DSM14241), was reported to improve markers of oxidative stress, such as total antioxidative activity (TAC) or glutathione red/ox ratio in healthy volunteers [264]. These results prompted later studies with this and other strains of lactobacilli [265], demonstrating the ability of some of these microorganisms to uptake and reduce the oxidised glutathione from the environment, contributing to the GSH/GSSG balance [263,266]. Moreover, these observations promoted the study of the application of antioxidant lactobacilli in different conditions, with positive results in the reduction of inflammation and myeloperoxidase activity in an animal model of induced colitis [267] or, more recently, the improvement of vascular endothelial damage in a model of chronic nitric oxide (NO) synthase inhibition [268]. Different mechanisms have been proposed for these effects of lactobacilli administration, including the ability to reduce glutathione or to produce enzymes such as feruloyl esterases able to release antioxidant hydroxycinnamic acids from undigested dietary compounds [263,266,269]. In addition to the abovementioned mechanisms, the bacterial metabolites, mainly SCFA, may also play a role. Acetate and butyrate, two of the main SCFA produced in the colon, seem to be able to downregulate the expression of G protein-coupled receptors (GPRs), increasing NO and reducing the production of ROS, thus mediating an antioxidative effect [270]. To this regard, in a PD model, the administration of a probiotic mix increased the levels of butyrate and neurotrohic factors in the brain, attenuating induced neuronal loss in the nigrostriatal pathway [271].

The increasing interest in the development of intervention tools for the prevention of age-related diseases and neurodegeneration has led to an increasing number of studies aiming at reducing brain oxidative stress by using lactobacilli or other probiotics such as bifidobacteria. The number of human intervention studies in this area is still limited with some data suggesting a potentially beneficial role for probiotics in AD with reductions on the levels of a lipid peroxidation product such as malondialdehyde (MAD), a common marker of oxidative stress, without detecting differences in TAC or NO [211]. Similarly, human studies have also been conducted on Parkinson’s patients or persons with mild cognitive impairment. A recent systematic review on the topic [272] concludes that although probiotics must be promising more RCT trials are needed.

Therefore, to date, most evidence in this area comes from animal studies. Different animal models have been used in the different studies. For example, by using a D-Galactose-induced aging mice model, Li et al. [273] demonstrated that the administration of a *L. fermentum* strain was able to induce the expression of antioxidant enzymes in the brain through the activation of the Nrf2 signalling pathway. Similarly, *Lactiplantiibacillus plantarum* strain Dp189 was recently reported to prevent cognitive decline and the hyperfosforilation of tau protein in a similar model [274]. The same strain has also been shown to downregulate oxidative damage in a PD model [275]. Moreover, the administration of *L. plantarum* GKM3 to senescence-accelerated mice (SAMP8) was found to reduce the bran levels of two oxidative markers: the 8-hydroxy-2′-deoxyguanosine and the lipid peroxidation by-products TBARS [273]. It is important to point out that, although the most studied, lactobacilli are not the only probiotics that have been tested. Bacterial mixes, also containing bifidobacterial strains, have also been reported to pose beneficial effects by reducing MDA and SOD levels in a β-amyloid injection model [276] or by limiting neuronal damage and lipid peroxidation in a 6-hydroxydopamine-induced PD model [277]. Not just bacteria but also the yeast *Saccharomyces boulardii*, a commonly used probiotic microorganism, has demonstrated a positive effect on brain oxidative stress in a mice model of antibiotic-induced dysbiosis [278]. The administration of this yeast was found to reduce MDA levels and acetylcholine esterase and myeloperoxidase activities in the brain whist it increased GSH and SOD activity.

## 8. Conclusions

Multiple lines of evidence provide strong support for the involvement of oxidative stress in relation to PA. Moreover, the dual role of oxidative stress in essential neuroprotective cellular mechanisms versus detrimental effects of increased uncontrolled ROS production should be carefully considered while developing strategies to mitigate oxidative stress under PA conditions. Indeed, we have discussed how the preventative and therapeutic effects of exercise are associated with the modality as well as the duration and intensity. Besides, moderate to vigorous intensity, along with the long duration and high frequency of exercise, has better benefits. The positive impacts of exercise on brain health might be associated with an improved mitochondrial function, enhanced production of BDNF, increased cerebral perfusion, and neurogenesis.

Moreover, we want to point out the relevance of the microbiota–gut–brain axis, which links the health of the gut microbiota and the central nervous system. Any imbalance in the commensal gut microbiota leads to aberrant endocrine, immunological, and neuronal signals that ultimately harm neuronal development and aggravate the age-related and neurodegenerative disease’s symptoms. Biotherapy using “psycobiotics” shows immense potential as therapeutic or prophylactic agents against oxidative stress and, also, due to their role to reinstate balance to the microbiota and the corresponding pathways that link microbial metabolism and brain functions.

## Data Availability

No new data were created or analysed in this study. Data sharing is not applicable to this article.
